# Silver-coated silicone stents as an approach to prevent bacterial colonization of central airways after tracheobronchial stenting

**DOI:** 10.3389/fmicb.2025.1713806

**Published:** 2025-12-03

**Authors:** Rosa López-Lisbona, Laura Calatayud, Joan Gilabert-Porres, Marta Díez-Ferrer, Salvador Borros, Carmen Ardanuy, Salud Santos, Antoni Rosell, Sara Martí

**Affiliations:** 1 Pulmonology Department, Hospital Universitari de Bellvitge, IDIBELL, Hospitalet de Llobregat, University of Barcelona, Barcelona, Spain; 2 Microbiology Department, Hospital Universitari de Bellvitge, IDIBELL, Hospitalet de Llobregat, University of Barcelona, Barcelona, Spain; 3 CIBER de Enfermedades Respiratorias (CIBERES), Instituto de Salud Carlos III, Madrid, Spain; 4 Institut Quimic Sarria, Universitat Ramon Llull, Barcelona, Spain; 5 Pulmonology Department, Hospital Universitari Germans Tries i Pujol, Universitat Autonoma Barcelona, Barcelona, Spain

**Keywords:** silver-coating, tracheobronchial stents, *Pseudomonas aeruginosa*, *Staphylococcus aureus*, bacterial colonization

## Abstract

**Rationale:**

Tracheobronchial stents are used to treat central airway obstruction but frequently develop bacterial colonization that can lead to infection.

**Objectives:**

To identify the microorganisms responsible for stent colonization and to evaluate the *in vitro* ability of silver-coated silicone to reduce their growth.

**Methods:**

Bacterial identification and quantification were performed on bronchial washings obtained prospectively from 30 patients before and at the first follow-up after stent placement. Bacterial viability on silver-coated silicone was determined for six clinical isolates and two collection-type strains by confocal microscopy and counts of bacterial colony-forming units.

**Measurement and main results:**

The most frequently recovered pathogens were *Pseudomonas aeruginosa* (30%) and *Staphylococcus aureus* (23%). An increase in bacterial load of colonizing pathogens was observed at the first follow-up after stenting, with long-term persistence of the same bacterial genotype within those patients. Three *P. aeruginosa* and three *S. aureus* clinical isolates were selected to evaluate the effect of silver on bacterial colonization. Silver-coated silicone exhibited high bactericidal activity against all isolates tested, with bacterial death ranging from 88 to 96% for *P. aeruginosa* and from 77 to 88% for *S. aureus*.

**Conclusion:**

Silver-coated silicone significantly reduced the viability of the most common pathogens that colonized tracheobronchial stents and may represent a promising option for preventing stent-related infections.

## Introduction

Central airway obstruction associated with malignant and nonmalignant processes can cause a clinical spectrum that ranges from mild shortness of breath to respiratory failure, and can lead to patient mortality (1, 2). Airway stenting is an established method for prolonging survival and offering symptomatic palliative relief of large airway obstruction in advanced carcinoma patients (2–6). In nonmalignant disease, such as post-intubation trauma, granulomatosis or tuberculosis, stents are commonly used in patients with complex lesions or comorbidities (7, 8) and may be used as a bridge awaiting the appropriate curative treatment (8, 9).

Airway stents can be made of silicone, nitinol (nickel-titanium alloy), or a hybrid that combines a metallic framework with a silicone body (1, 10–13). Since 2005, the U.S. Food and Drug Administration (FDA) has advised that metallic stents should not be the first choice for patients with benign disease because they often become embedded in the airway tissue (14). Given that the primary goal for patients with benign disease is to achieve a stent-free airway (15), silicone stents are preferred for improving respiratory symptoms because they are easily removed or replaced (1, 10–12, 16–18). As a result, metallic stents are generally reserved as permanent palliative treatment in malignant airway obstruction (6, 12). Nevertheless, a new generation of fully covered metallic stents has been developed and marketed since the FDA warning (12, 13, 19, 20). These newer stents have shown promising outcomes in nonmalignant disease (21), can also be safely removed in selected patients with malignant disease who respond well to treatment and even may have good results in benign airways stenosis (21–23). Despite the improvement in symptoms and quality of life, airway stents are associated with complications such as migration, granulation tissue formation or mucous plugging, which occur in approximately 15–42% of cases (3, 24, 25).

Bacterial colonization is a common issue observed after airway stenting. The insertion of a foreign object in the airway can induce mucostasis, which serves as a culture medium for colonizing bacteria (11, 25, 26). This colonization may lead to halitosis, mucus plugging, infection or even sepsis (27), complications that can require additional bronchoscopies or early stent replacement, resulting in increased costs (28, 29) and patient dissatisfaction. Clinicians should place greater emphasis on active surveillance of central airway colonization prior to stent placement as a strategy to prevent infection. Basic measures such as adequate patient hydration, saline nebulization, effective coughing and routine bronchoscopic monitoring are essential; however, colonization may persist despite these efforts (29). A reduction in pathogenic bacterial colonization has already been demonstrated with silver-coated endotracheal tubes in intubated patients in Intensive Care Units (30). Based on this, our aim was to retrospectively identify and quantify the microorganisms involved in stent colonization, assess the presence of potentially pathogenic bacteria in bronchial washings (BW) obtained before and after stenting, and prospectively evaluate the effect of silver-coated silicone as a mechanism for reducing bacterial growth and adherence by clinically relevant isolates.

## Materials and methods

### Study design and sample collection

We conducted a retrospective study of patients who underwent airway stent implantation at Hospital Universitari de Bellvitge (HUB, Barcelona, Spain) over a 40-month period, with prospective collection of clinical samples. BW samples were obtained in the Pulmonology Department as part of routine clinical practice, both prior to stenting and during the first bronchoscopy follow-up after stent placement. Samples were sent to the Microbiology Department for further analysis. Informed consent was not required as the microbiological evaluation was part of standard care. Patient confidentiality was preserved in accordance with national regulations. This manuscript was reviewed for publication by the Clinical Research Ethics Committee of Bellvitge University Hospital (PR186/14).

### Stent implantation

Patients who met established clinical criteria for airway stenting, as defined by international guidelines (31) were included in the study. Endotracheal intubation was performed under general anesthesia using an EFER-Dumon® rigid bronchoscope (EFER Endoscopy, La Ciotat, France), and flexible bronchoscopy was used to obtain BW samples. When indicated, treatment with a neodymium-aluminum-perovskite (Nd-YAP) laser (Lokki, Lobel Medical SAS, The Rocks Condrieu, France) was applied. Airway stenting was performed using a prosthesis selected, according to the type of stenosis (31, 32), including Dumon® (Novatech SA, Grasse, France), Ultraflex® (Boston Scientific, Natick, MA, USA) or Aerstent® (Leufen Medical-BESS, Aachen, Germany) stents. No significant complications were reported during the procedure.

### Microbiological analysis

Samples were processed within 24 h of collection. Firstly, samples quality was screened by Gram Stain and only good-quality samples, according to the criteria described by Domenech et al. were considered (<10 squamous cells and >25 leukocytes per low-power field) for culture (33). BW samples were homogenized with dithiothreitol (Sputolysin, Oxoid), serially diluted (1:10^−1^, 1:10^−2^, 1:10^−3^) and plated onto blood, chocolate and MacConkey agar. Plates were incubated overnight at 37 °C (blood and chocolate at 5% CO2; MacConkey in ambient air). After incubation, colony-forming units per milliliter (CFU/mL) were calculated and sub-cultured for bacterial identification by standard microbiology methods (33, 34). The following ranges were used: <10^2^ CFU/mL was considered negative, ≥10^2^ to <10^6^ CFU/mL was considered low-grade colonization, and ≥10^6^ CFU/mL indicated high-grade colonization (35). Bacterial isolates were classified as Potential Pathogenic Microorganisms (PPMs) and non-PPMs, according to previously described (36).

### Molecular genotyping and antimicrobial susceptibility

Molecular genotyping was determined by Pulse Field Gel Electrophoresis (PFGE) with the restriction enzymes *Spe*I (*P. aeruginosa*) and *Sma*I (*S. aureus*) following the manufacturer’s directions (New England BioLabs, Ipswich, MA, USA). Fragments were separated using a CHEF-DRIII system (BioRad), as previously described (37). PFGE band patterns were analyzed using the Fingerprinting II Software 3.0 (BioRad). The similarity of the PFGE banding patterns was estimated with the Dice coefficient, setting the optimization and tolerance at 1%. Isolates with ≥ 85% relatedness were considered highly genetically related.

Antimicrobial susceptibility was determined by microdilution for *P. aeruginosa* and by disc diffusion for *S. aureus*, following the recommendations of the Clinical Laboratory Standards Institute (CLSI) (38). *P. aeruginosa* was tested against ticarcillin, piperacillin/tazobactam, ceftazidime, cefepime, aztreonam, gentamicin, tobramycin, amikacin, ciprofloxacin, levofloxacin, imipenem, meropenem and colistin; *S. aureus* was tested against penicillin, oxacillin, cefoxitin, erythromycin, clindamycin, trimethoprim/sulfamethoxazole, chloramphenicol, gentamicin, tobramycin, tetracycline, rifampicin, ciprofloxacin and vancomycin.

### Silver-coated silicone

Silver was deposited onto polydimethylsiloxane (PDMS), a silicone based organic polymer, by a chemical solution method. PDMS films were fabricated following the general protocol previously described (39) using the Sylgard 184 silicone elastomer kit (Ellsworth Adhesives, Spain), mixed at a 10:1 base-to-curing-agent ratio. The mixture was cast on a glass substrate using a paint applicator to form a 500 μm thick layer, cured at 60 °C overnight, and then cut into circulars disks (21 mm in diameter). Surface modifications were performed in a custom-built RF plasma reactor (13.56 MHz) previously described (39, 40). PDMS samples were placed planar to the electrode to ensure that only the upper surface was exposed to the plasma. Penta-fluorophenyl methacrylate (PFM) vapor was introduced at a working pressure of 0.02–0.04 mbar in the reactor chamber and plasma polymerized (15 W, 10/10 duty cycle for 5 min) to form a thin reactive pp-PFM (plasma polymerized PFM) coating followed by 3 min of monomer flow exposure (post-polymerization time). More information about plasma reactor and plasma polymerization process can be found in Supplementary material.

Silver was subsequently generated *in situ* through a Tollen’s reaction (41) adapted from previous work (39, 40). Briefly, glucosamine was first grafted to the pp-PFM modified PDMS surface (1 M solution, pH 7.4 Incubated during 6 h) and acted as a mild carbohydrate-based reducing agent enabling the controlled reduction of silver ions to metallic silver. More information about the silver deposition process can be found in Supplementary material.

To determine the total silver content, the metallic coating was completely dissolved in concentrated nitric acid (Sigma-Aldrich, Burlington, Massachussetts, United States), and the resulting solution was diluted with Mili-Q water prior to analysis. The silver concentration was quantified using inductively coupled plasma-optical emission spectroscopy (ICP-OES, Optima 2,100 DV, PerkinElmer) (39, 40). The resulting silver-coated films had a silver concentration of 0.6 ± 0.3 μg/mm^2^.

### Bacterial adhesion assay

Overnight bacterial cultures were diluted in fresh tryptone-soy broth (TSB) (Oxoid, Thermo Fisher Scientific, Basingstoke, UK) to an OD_600_ of 0.1. Sterile and silver-coated PDMS slides were placed into 24-well plates (Nunc, Thermo Fisher Scientific, Roskilde, Denmark), covered with 1 mL of the previously adjusted culture, and incubated for 24 h at 37 °C without shaking. At this point, the slides were used to compare bacterial viability between treated and untreated samples by confocal microscopy after live/dead staining or by determination of the CFU/mL.

### Bacterial viability

Clinical isolates from the prospective samples were used to assess bacterial adhesion and compared to the type strains: *P. aeruginosa* PAO1 and *S. aureus sub*sp. *aureus* (ATCC® 29213™). Bacterial viability was studied by confocal microscopy and CFU quantification.

#### Confocal microscopy

Slides were washed and stained with the Live/Dead® BacLight™ Bacterial Viability Kit (Life Technologies, Madrid, Spain), following the manufacturer’s instructions. Images were randomly acquired using a Leica TCS-SL filter-free spectral confocal laser-scanning microscope (Leica Microsystems, Manheiem, Germany) and analyzed with the Leica Confocal Software 2.5 (Leica Microsystems, Mannheim, Germany). Bacterial viability was calculated with the ImageJ software.^1^ Additional methodological details are available in the Supplementary material.

#### Bacterial CFU quantification

Slides were washed twice by decantation, placed in tubes (Eppendorf, Hamburg, Germany) containing 500 μL of 0.9% NaCl (Sigma-Aldrich, St. Louis, MO, USA), and vortexed for 10 min to detach adherent bacteria. Serial dilutions were performed on the homogenized suspensions, which were plated onto blood agar plates (Becton Dickinson, Franklin Lakes, NJ, USA) and incubated at 37 °C. The CFU count per milliliter was calculated after 24 h of growth.

### Statistical analysis

Statistical analysis was performed with the GraphPad Prism 5 software (GraphPad Software, Inc., Dotmatics California, USA). Differences were evaluated using the Fisher exact test or the chi-squared test with Yates’ correction. A *p* value less than 0.05 was considered statistically significant. Means ± standard errors of the means for at least three independent replicates are reported. One-way analysis of variance with the Newman–Keuls multiple-comparison *post hoc* test was used for statistical analysis when appropriate.

## Results and analysis

### Clinical characteristics

A total of 30 patients with benign and malignant central airway disease were included during the study period. The clinical characteristics of the patients are summarized in Table 1. The mean age was 63 years (43–79), and most of the patients (87%) were male. Silicone stents were used in most cases (73%), followed by metallic stents (17%) or a combination of both silicone and metallic stents (10%). Stents were placed in the trachea (20%), bronchi (37%), or both locations (tracheobronchial, 43%). The main indication for stent placement was malignancy (97%); only one patient had a benign stenosis. No signs or symptoms of bacterial infection were observed in any patient with a median follow-up of 33 days (IQR 21–36) after stenting.

**Table 1:** Clinical characteristics of the 30 patients with airway stents (*n* = 30).

Patient	Gender	Age	Smoker	Type stent	Endobronchial lesion	Cancer	Stent location	Immunosuppression
1	Male	74	Ex	Silicone	Infiltration	Thyroid	Trachea	No
2	Male	69	Current	Silicone	Infiltration	Lung	Bronchial	Yes
3	Male	72	Current	Silicone	Extrinsic compression	Lung	Bronchial	No
4	Female	62	No	Silicone	Benign stenosis	–	Trachea	No
5	Female	58	Ex	Silicone + Metallic	Infiltration	Lung	Bronchial	Yes
6	Male	68	Ex	Silicone	Infiltration	Thyroid	Trachea	No
7	Male	66	Ex	Metallic	Infiltration	Lung	Bronchial	Yes
8	Male	65	Current	Silicone	Infiltration	Lung	Tracheobronchial	No
9	Male	66	Current	Silicone	Infiltration	Lung	Tracheobronchial	No
10	Male	70	Current	Silicone	Infiltration	Lung	Trachea	No
11	Male	61	Ex	Silicone	Extrinsic compression	Thyroid	Trachea	No
12	Male	55	Ex	Silicone	Infiltration	Lung	Tracheobronchial	Yes
13	Male	61	Current	Silicone	Infiltration	Lung	Tracheobronchial	No
14	Male	49	Ex	Metallic	Fistula	Esophagus	Trachea	No
15	Male	75	Ex	Silicone	Endobronchial tumor	Lung	Bronchial	No
16	Male	66	Current	Silicone	Infiltration	Lung	Tracheobronchial	No
17	Male	54	No	Silicone + Metallic	Infiltration	Skin	Bronchial	Yes
18	Female	77	No	Silicone	Infiltration	Ovary	Bronchial	No
19	Male	64	Ex	Silicone	Infiltration	Lung	Tracheobronchial	Yes
20	Male	43	Current	Silicone	Infiltration	Lung	Tracheobronchial	No
21	Male	79	Ex	Silicone	Infiltration	Colon	Tracheobronchial	Yes
22	Female	44	Current	Silicone	Infiltration	Lung	Tracheobronchial	Yes
23	Male	58	Current	Metallic	Infiltration	Lung	Bronchial	Yes
24	Male	56	Current	Silicone	Infiltration	Lung	Tracheobronchial	Yes
25	Male	66	Ex	Silicone/Metallic	Infiltration	Lung	Tracheobronchial	No
26	Male	59	Ex	Metallic	Infiltration	Lung	Bronchial	No
27	Male	66	Current	Silicone	Infiltration	Lung	Bronchial	No
28	Male	77	Ex	Silicone	Infiltration	Lung	Tracheobronchial	No
29	Male	52	Ex	Silicone	Infiltration	Kidney	Tracheobronchial	Yes
30	Male	63	Current	Metallic	Infiltration	Lung	Bronchial	Yes

### Isolation of potentially pathogenic bacteria

#### Bacterial identification and quantification

Of the 60 BW samples submitted for microbial analysis, significant bacterial counts were observed in 46 samples (77%). In the remaining 14 samples, no microorganisms exceeded the threshold of >10^2^ CFU/mL. Low levels of bacterial colonization (≤10^6^ CFU/mL) were observed in 18 samples (30%), while high colonization levels (>10^6^ CFU/mL) were found in 28 samples (47%).

A total of 42 PPMs were isolated from 33 BW samples obtained from 23 patients. *P. aeruginosa* (11 BW samples from nine patients) and *S. aureus* (10 BW samples from six patients) were the most frequently detected microorganisms. Seven patients with PPMS in the BW had the same isolation in both samples (23%), being *P. aeruginosa* the same microorganism in two patients and *S. aureus* in four patients. Table 2 shows the microbial pathogens isolated among the 60 BW samples collected before and after stent implantation, together with the bacterial quantification (CFU/mL) within the samples. High bacterial loads of *P. aeruginosa* (range: 1.2 × 10^6^ to 1.8 × 10^9^ CFU/mL) were found in nine samples, and of *S. aureus* (range: 2.2 × 10^7^ to 2.4 × 10^8^ CFU/mL) in six samples. Patients colonized with the same PPM before and after stenting tended to show an increased bacterial load at the first follow-up after stent placement (five of seven patients, 71%).

**Table 2:** Microbial pathogens isolated from bronchial washings before and after tracheobronchial stent placement.

Patient	Days^a^	CFU/mL	Microorganism^b^
1	baseline	3·10^4^	***Streptococcus pneumoniae***
	**40d**	1·10^8^ + 2·10^7^ + 3·10^7^	***Pseudomonas aeruginosa (P3)*** + *Candida albicans* + ***Klebsiella oxytoca***
2	baseline	5·10^5^	Viridans Group Streptococci
	**33d**	6·10^4^ + 6·10^4^ + 4·10^2^	***Pseudomonas aeruginosa (P3)*** + Coagulase negative staphylococci + *Candida albicans*
3	baseline	2·10^6^ + 1·10^5^	***Pseudomonas aeruginosa (P1)*** + ***Streptococcus pneumoniae***
	**35d**	1.2·10^6^	***Pseudomonas aeruginosa (P1)***
4	baseline	1.6·10^7^	Viridans Group Streptococci
	**67d**	4·10^7^ + 5.2·10^7^	***Pseudomonas aeruginosa (P3)*** + Viridans Group Streptococci
5	baseline	2.4·10^5^	***Staphylococcus aureus (S2)***
	**33d**	6·10^6^ + 2.2·10^7^ + 1·10^5^	***Enterobacter cloacae*** + ***Staphylococcus aureus (S2)*** + ***Stenotrophomonas maltophilia***
6	baseline	8·10^4^	Viridans Group Streptococci
	**18d**	8·10^6^ + 2·10^5^	***Citrobacter koseri*** + Viridans Group streptococci
7	baseline	4·10^5^	Viridans Group Streptococci
	**29d**	2.8·10^6^ + 2·10^6^ + 2·10^4^	***Enterobacter aerogenes*** + Viridans Group Streptococci + *Candida albicans*
8	baseline	1.6·10^3^	Viridans Group streptococci
	**15d**	2·10^7^ + 2·10^6^	***Enterobacter cloacae*** + Viridans Group Streptococci
9	baseline	1·10^3^	***Staphylococcus aureus (S2)***
	**34d**	2.4·10^8^ + 8·10^7^	***Staphylococcus aureus (S2)*** + *Corynebacterium* sp.
10	baseline	4·10^4^ + 2·10^6^	Viridans Group Streptococci + *Corynebacterium* sp.
	**35d**	3·10^5^ + 6·10^4^ + 2·10^3^	***Moraxella catharralis*** + *Corynebacterium* sp. + Viridans Group Streptococci
11	baseline	Negative	
	**14d**	4·10^7^	***Staphylococcus aureus (S3)***
12	baseline	6·10^5^	Viridans Group Streptococci
	**15d**	Not significant (<10^2^)	
13	baseline	1·10^3^	***Haemophilus influenzae***
	**40d**	3·10^7^	***Pseudomonas aeruginosa (P6)***
14	baseline	9·10^5^ + 6·10^2^	*Acinetobacter lwofii* + ***Stenotrophomonas maltophilia***
	**22d**	8·10^5^ + 4·10^2^	***Stenotrophomonas maltophilia*** + ***Escherichia coli***
15	baseline	4.4·10^3^	Viridans Group Streptococci
	**14d**	6.4·10^7^	***Staphylococcus aureus (S4)***
16	baseline	Negative	
	**27d**	1.1·10^8^	***Pseudomonas aeruginosa (P2)***
17	baseline	2.8·10^7^	***Staphylococcus aureus (S1)***
	**16d**	2·10^4^ + 1.4·10^5^	***Staphylococcus aureus (S1)*** + ***Enterobacter cloacae***
18	baseline	2·10^7^	***Enterobacter cloacae***
	**329d**	Not significant (<10^2^)	
19	baseline	6·10^2^	*Staphylococcus epidermidis*
	**17d**	1·10^9^	Viridans Group Streptococci
20	baseline	Not significant (<10^2^)	
	**22d**	Not significant (<10^2^)	
21	Baseline	Not significant (<10^2^)	
	**36d**	3·10^8^ + 3·10^7^	***Pseudomonas aeruginosa (P7)*** + ***Serratia marcescens***
22	Baseline	Negative	
	**43d**	Not significant (<10^2^)	
23	Baseline	2·10^2^	**Methicilin resistant *Staphylococcus aureus (S3)***
	**34d**	1·10^8^ + 6·10^6^ + 2·10^2^	**Methicilin resistant *Staphylococcus aureus (S3)*** + *Neisseria* spp. + ***Enterobacter cloacae***
24	Baseline	6·10^4^ + 3·10^5^ + 8·10^5^	***Pseudomonas aeruginosa (P4)*** + Viridans Group Streptococci + *Corynebacterium* sp.
	**36d**	1.8·10^9^ + 1.6·10^9^	***Pseudomonas aeruginosa (P4)*** + ***Enterobacter aerogenes***
25	Baseline	1·10^8^ + 3·10^7^	***Serratia marcescens*** + *Streptococcus parasanguis*
	**28d**	6.4·10^8^	***Proteus mirabillis***
26	Baseline	Not significant (<10^2^)	
	**28d**	6·10^8^	***Pseudomonas aeruginosa (P5)***
27	Baseline	4·10^8^	***Streptococcus pneumoniae***
	**36d**	1.6·10^7^ + 6·10^7^	*Staphylococcus lugdunensis* + *Corynebacterium* sp.
28	Baseline	Not significant (<10^2^)	
	**21d**	Not significant (<10^2^)	
29	Baseline	Negative	
	**43d**	1.6·10^8^	*Corynebacterium* sp.
30	Baseline	4·10^6^ + 1.6·10^5^	Viridans Group Streptococci + *Rothia mucilaginosa*
	**48d**	Not significant (<10^2^)	

Data represent the results of 60 samples from 30 patients. ^a^Underlined, sample taken before stenting (baseline); bold, sample taken after stenting. ^b^Bold, potential pathogenic microorganisms (PPMs).

#### Bacterial acquisition after stenting

Before stenting, bacterial colonization was present in 22 out of 30 patients (73%), with 13 PPMs isolated from 12 of these patients. After stenting, 24 out of 30 patients (80%) were colonized, with a median follow-up of 33 days (IQR 21–36), with 29 PPMs identified from 21 patients (Table 3). Differences were observed in the isolation of PPMs before and after stenting, with *P. aeruginosa* being the main microorganism recovered from BW samples after stenting (9 of 11 samples, 82%), followed by *Enterobacter cloacae* (4 of 5 samples, 80%), and *S. aureus* (6 of 10 samples, 60%). *P. aeruginosa* was also the main pathogen associated with stenting, with seven non-colonized patients acquiring the organism after the procedure.

**Table 3:** Microbial pathogens isolated.

	Number of samples (%) (*N* = 74)	Pre-stenting BW^a^		Post-stenting BW^b^	
No (%)		Bacterial load (CFU/mL)	No (%)	Bacterial load (CFU/mL)
**Potential Pathogenic Bacteria**	**42 (57%)**				
*Pseudomonas aeruginosa*	11 (15%)	2 (18%)	6·10^4^–2·10^6^	9 (82%)	6·10^4^–1.8·10^9^
*Staphylococcus aureus*	10 (13,5%)	4 (40%)	2·10^2^–2.8·10^7^	6 (60%)	2·10^4^–2.4·10^8^
*Streptococcus pneumoniae*	3 (4%)	3 (100%)	3·10^4^–4·10^8^	0	–
*Stenotrophomonas maltophilia*	3 (4%)	1 (33%)	6·10^2^	2 (67%)	1·10^5^–8·10^5^
*Moraxella catarrhalis*	1 (1.3%)	0	–	1 (100%)	3·10^5^
*Haemophilus influenzae*	1 (1.3%)	1 (100%)	1·10^3^	0	–
*Enterobacteriaceae*					
*Enterobacter cloacae*	5 (6.8%)	1 (20%)	2·10^7^	4 (80%)	2·10^2^–2·10^7^
*Enterobacter aerogenes*	2 (3%)	0	–	2 (100%)	2.8·10^6^–1.6·10^9^
*Serratia marcescens*	2 (3%)	1 (50%)	1·10^8^	1 (50%)	3·10^7^
*Citrobacter koseri*	1 (1.3%)	0	–	1 (100%)	8·10^6^
*Escherichia coli*	1 (1.3%)	0	–	1 (100%)	4·10^2^
*Klebsiella oxytoca*	1 (1.3%)	0	–	1 (100%)	3·10^7^
*Proteus mirabilis*	1 (1.3%)	0	–	1 (100%)	6.4·10^8^
**Non-Pathogenic Bacteria**	**32 (43%)**				
Viridans Group Streptococci	17 (23%)	11 (65%)	1.6·10^3^–3·10^7^	6 (35%)	2·10^3^–1·10^9^
*Corynebacterium* spp.	6 (8%)	2 (33%)	8·10^5^–2·10^6^	4 (67%)	6·10^4^–1.6·10^8^
*Candida albicans*	3 (4%)	0	–	3 (100%)	4·10^2^–2·10^7^
Coagulase negative Staphilococci	3 (4%)	1 (33%)	6·10^2^	2 (67%)	6·10^4^–1.6·10^7^
*Neisseria* spp.	1 (1.3%)	0	–	1 (100%)	6·10^6^
*Acinetobacter lwofii*	1 (1.3%)	1 (100%)	9·10^5^	0	–
*Rothia mucilagenosa*	1 (1.3%)	1 (100%)	1.6·10^5^	0	–

Data represent the results of 60 samples from 30 patients.

^a^Number of isolates (percentage) recovered from pre-stenting samples; ^b^number of isolates (percentage) recovered from post-stenting samples.

### Molecular genotyping

Molecular genotyping was performed by PFGE on the two most frequently isolated PPMs: *P. aeruginosa* and *S. aureus*. Isolates from patients with more than one sample (pre- and post-stenting) were genotypically identical. Two patients carrying a *P. aeruginosa* strain and four patients with *S. aureus* before stenting conserved the same strain at the first follow-up, including a methicillin resistant *S. aureus* (MRSA) isolate.

Eleven *P. aeruginosa* isolates were separated into seven independent genotypes. Isolates obtained within a short time frame from three patients (patients 1, 2 and 4) shared the same PFGE pattern. Similarly, ten *S. aureus* isolates were separated into four independent genotypes. Four isolates from two patients, collected at the same time (genotype S2) and three isolates from two patients recovered within a 19-month period (genotype S3) shared the same PFGE pattern.

Routine follow-up revealed persistence of the same *P. aeruginosa* genotype in seven patients for periods ranging from 36 to 448 days, and of the same *S. aureus* genotype in five patients from 35 to 182 days (data not shown).

### Antimicrobial susceptibility

Bacterial isolates recovered before and after stent placement were genotypically identical and maintained the same antimicrobial susceptibility profile. Therefore, only one sample per patient was included in the analysis. Bacterial susceptibility was high among all the PPMs recovered.

*P. aeruginosa* strains showed susceptibility to ceftazidime, aminoglycosides and quinolones, with only the isolates from one patient presenting resistance to those antimicrobial groups (i.e., a multi-drug resistant isolate susceptible only to amikacin and colistin). All the isolates were susceptible to colistin.

Of the six *S. aureus* isolates, five were methicillin-susceptible and half (3/6) were resistant to ciprofloxacin. Only one isolate was resistant to methicillin and had additional resistance to erythromycin and ciprofloxacin.

Additional information regarding the antimicrobial susceptibility profiles has been included in Supplementary Table S1 (Excel file).

### Bacterial viability

Six clinical isolates from the prospective samples were used to determine bacterial adhesion (Table 4) and compared to the type strains: *P. aeruginosa* PAO1 and *S. aureus sub*sp. *aureus* (ATCC® 29213™).

**Table 4:** Clinical and microbiological characteristics of the isolates selected for the bacterial adhesion assay.

Strain	Type stent	Stent location	Indication for stenting	CFU/mL	No samples^a^	Days^b^	Source
PAER 939	Silicone	Tracheobronchial	Malignancy	2·10^7^	4	51	This study
PAER 478	Silicone	Trachea	Malignancy	1·10^8^	5	486	This study
PAER 752	Silicone	Trachea	Benign stenosis	1·10^7^	3	141	This study
SAUR 151	Metallic	Bronchial	Malignancy	1·10^9^	3	73	This study
SAUR 014	Silicone + Metallic	Bronchial	Malignancy	2,8·10^7^	7	182	This study
SAUR 528	Silicone + Metallic	Bronchial	Malignancy	1,16·10^8^	3	105	This study

^a^Number of samples recovered from the patient with the same isolate; ^b^number of days the patient was colonized by the isolate.

Two control strains were used for the bacterial adhesion experiments: Control Strains: *P. aeruginosa* PAO1 (60); *S. aureus* ATCC® 29213™ (www.atcc.org).

**Figure 1:**
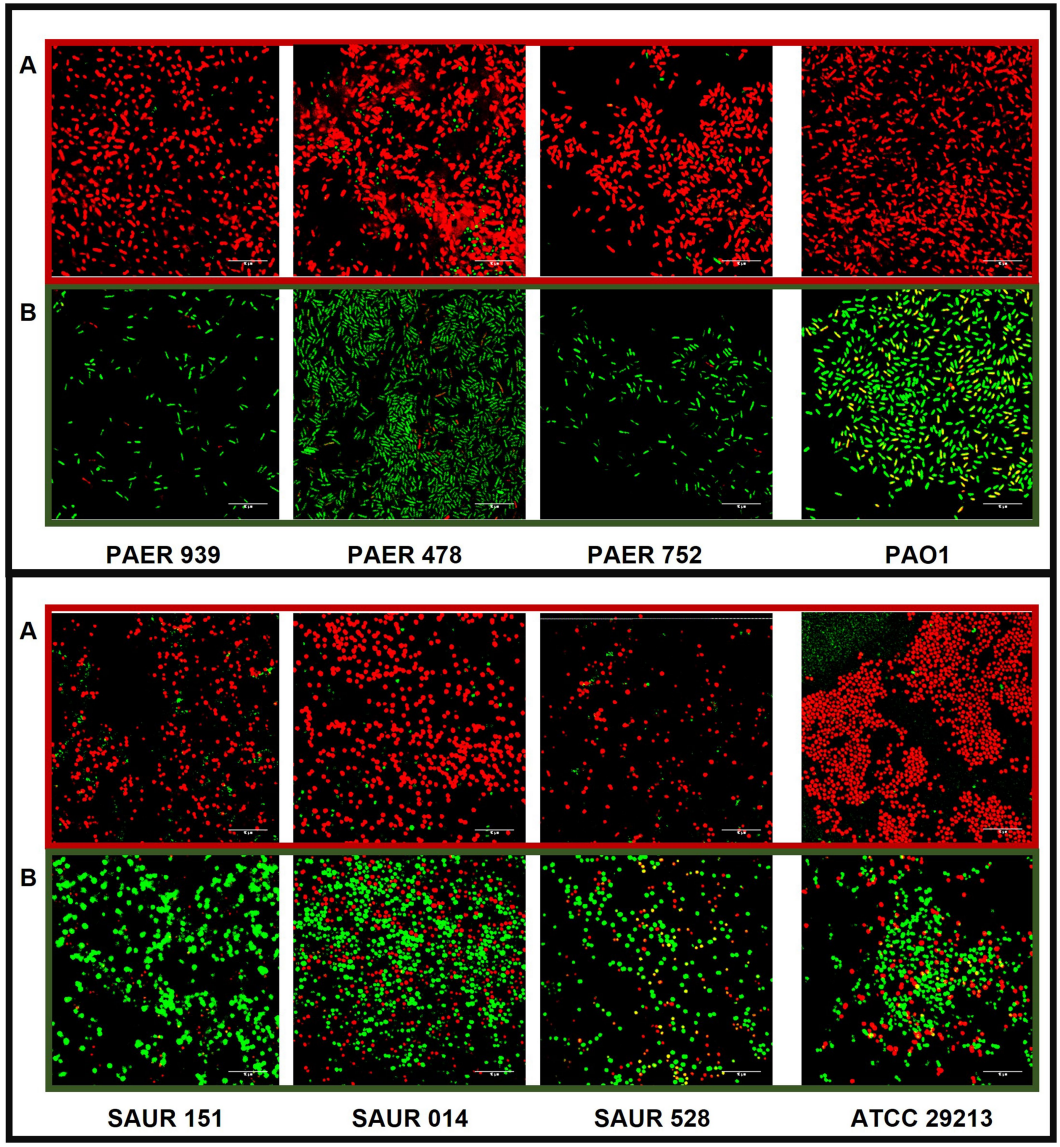
Representative images of live/dead staining of *P. aeruginosa* (top) and *S. aureus* (bottom) obtained by confocal microscopy. Dead bacteria are red and live bacteria are green. The scale bar represents 10 μm. Images of bacteria grown on silver-coated PDMS **(A)** and untreated PDMS **(B)**. PDMS, polydimethylsiloxane.

Bacterial colonization of both untreated PDMS and silver-coated PDMS slides was visualized by confocal microscopy after staining with a commercial live/dead assay. All bacteria were stained with the green Syto®9 dye (membrane permeable): only non-viable bacteria absorbed propidium iodide and were visualized in red (membrane impermeable dye). In the untreated PDMS slides, surface visualization by confocal microscopy showed that most of the attached bacteria were viable (green), in contrast to silver-coated PDMS slides, where the majority appeared non-viable (red) (Figure 1).

**Figure 2:**
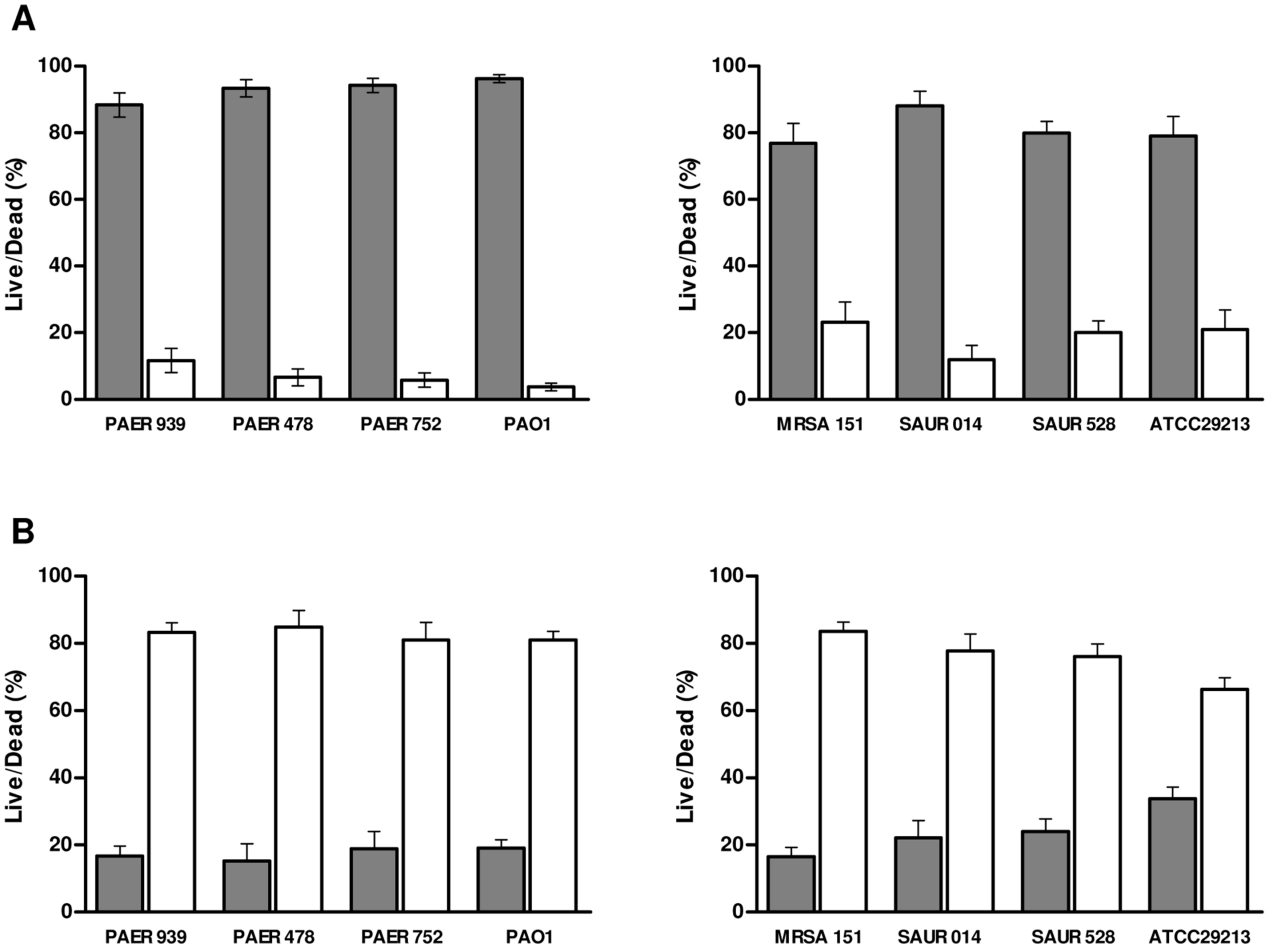
Bacterial viability on silver-coated **(A)** compared with uncoated PDMS **(B)** slides. Viability was expressed as the percentage of dead (grey) and alive (white) bacteria determined by confocal microscopy after live/dead staining. The data shows a clear reduction in bacterial viability on silver-coated surfaces for all isolates. Clinical isolates from patients with stent: PAER 939, PAER 478, PAER 752, MRSA 151, SAUR 014 and SAUR 528. Control Strains: *P. aeruginosa* PAO1 (60); *S. aureus* ATCC® 29213™. PDMS, polydimethylsiloxane.

Images were analyzed by separating the green and red fluorescence channels to quantify the surface area covered by live and dead bacteria. The percentage of dead bacteria was calculated from at least six microscopy images per condition (Figure 2), showing that the percentage of non-viable bacteria attached to silver-coated PDMS slides was higher (>75%) compared with the percentage of non-viable bacteria attached to untreated PDMS slides (<40%).

The bacterial viability of the clinical isolates and commercial strains was similar, although *P. aeruginosa* clinical isolates survived slightly better in silver than the reference PAO1 strain (Figure 2). Conversely, *P. aeruginosa* was more susceptible to silver treatment than *S. aureus*, with the percentage of bacterial death on silver-coated slides ranging from 88 to 96% for *P. aeruginosa*, compared with 77–88% for *S. aureus*.

**Figure 3:**
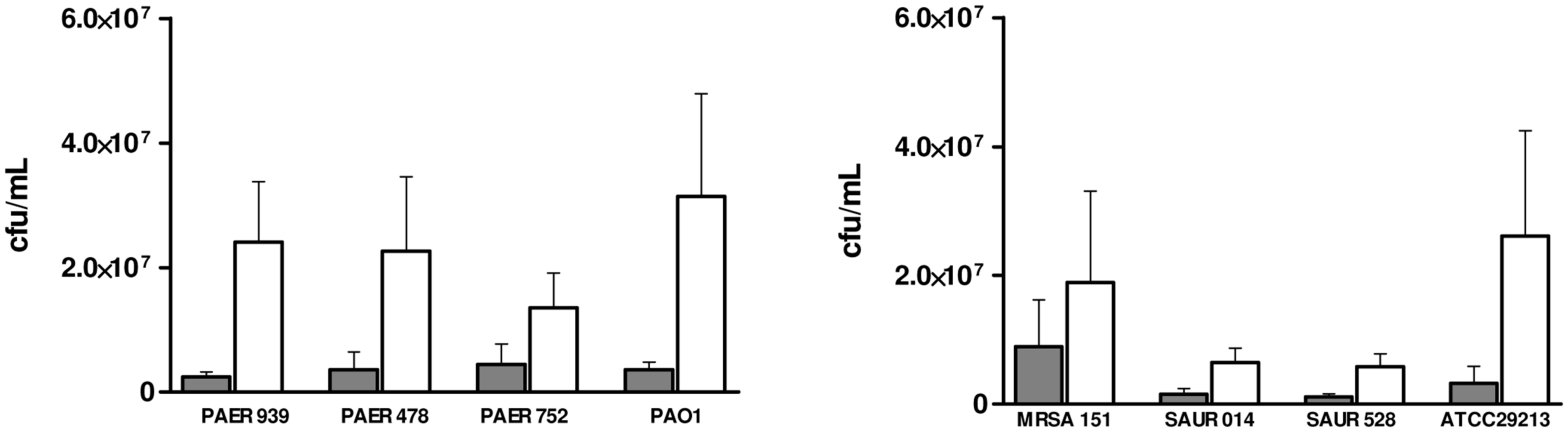
Bacterial viability on silver-coated (grey) compared with uncoated (white) PDMS slides defined by the colony-forming unit count per milliliter. Clinical isolates from patients with stent: PAER 939, PAER 478, PAER 752, MRSA 151, SAUR 014 and SAUR 528. Control Strains: *P. aeruginosa* PAO1 (60); *S. aureus* ATCC® 29213™. PDMS, polydimethylsiloxane.

These differences in bacterial viability were further supported by CFU/mL quantification on both untreated and silver-coated PDMS surfaces. Despite not being statistically significant due to differences in bacterial attachment to the untreated PDMS slides, we observed an important reduction in the number of bacteria growing on the silver-coated slides compared with the uncoated PDMS slides, for all the bacterial isolates (Figure 3).

## Discussion

Airway stenting is an established method for palliative or curative treatment of central airway obstruction, indicated to restore obstructed or weakened airways and to close fistulas (11, 27). Despite its many benefits, the introduction of a foreign body can lead to additional complications, including stent migration, obstruction, and colonization, the latter often leading to infection (11). Previous studies have reported colonization rates of PPMs of up to 78% after 1 month (11, 26), with stent-associated infections linked to increased mortality (39, 40).

Consequently, we conducted a retrospective study with prospective collection of clinical samples to implement bacterial colonization surveillance before and after stenting. Over a 3-year period, 30 patients met the inclusion criteria, and their pre- and post-stenting BW samples were recovered for microbiological analysis. To date, bacterial colonization in patients with airway stents has mostly been assessed in retrospective studies using previously collected data and samples (42–45). Although some prospective studies have been conducted (26, 42, 43), only one included both pre- and post-stenting bronchial samples from 14 patients (26). Our study was retrospective in design but included prospectively collected samples, allowing for more accurate comparison of colonization before and after stent placement in a larger patient cohort. To our knowledge, this study represents the largest analysis to date using prospectively collected bronchial samples to quantitatively assess bacterial colonization in patients undergoing airway stenting.

The main pathogens identified in our study were *P. aeruginosa* and *S. aureus*, corroborating findings from previous studies (26, 42, 43, 46). Most research has also found that *P. aeruginosa* was the main colonizing pathogen (26, 43, 47). Schmäl et al. identified *S. aureus* as the most frequent pathogen in Montgomery T-tubes; however, several stents were recovered from each patient, and only two patients were colonized by *S. aureus* (43% of the stents studied), while a third patient was co-colonized by both pathogens (42).

Both main pathogens showed a tendency to persist or increase their bacterial load from pre- and post-stenting. *P. aeruginosa* was the most prevalent pathogen, with 85% of the isolates presenting >10^6^ CFU/mL. Noppen et al. considered cutoff values of >10^3^ CFU/mL in protected specimen brush samples to be diagnostic of infection (26). Although no clinically significant infections were detected in our short-term follow-up, 83% of *P. aeruginosa* and 100% of *S. aureus* isolates exceeded this threshold (26). Colonization by PPMs increased from 40% before stenting to 70% after stenting, which represented a slightly higher colonization rate than previously shown by Noppen et al., who reported 36 and 50%, respectively (26). Our findings indicated that *P. aeruginosa* was the most frequently acquired pathogen within the first follow-up after stenting, while *S. aureus* showed no significant increase. In fact, it has been suggested that *P. aeruginosa* can adhere to tracheal epithelial cells even in patients without previous colonization (26, 36). Consequently, active surveillance and decolonization protocols before stenting may be effective to prevent *S. aureus* colonization but may be less effective against post-stent *P. aeruginosa* colonization.

Previous reports suggest that short-term systemic antimicrobial treatment may be ineffective in eradicating bacteria adhered to silicone stents, because of reduced susceptibility after adhesion occurs (42, 44). Nevertheless, bacteria within biofilms are far more resistant to treatment, indicating that standard susceptibility testing may not reflect *in vivo* behavior (45, 48). Biofilms are structured communities of microorganisms that strongly adhere to a solid surface and that are covered by a self-secreted protective layer that makes them more resistant to environmental stress (45, 48, 49). This condition can explain bacterial persistence despite high susceptibility of microorganisms to antimicrobials. In fact, we observed high persistence by the same bacterial genotypes, suggesting bacterial adherence to the stents.

Schmäl et al. proposed incorporating antimicrobial agents into silicone stents to reduce colonization and infection (42). Unfortunately, stent-coating with diffusible antimicrobial substances may be problematic if the minimum inhibitory concentration is not reached, and could even induce bacterial resistance (50, 51). Conversely, resistance to silver is rare and unlikely to develop, as silver attacks a broad range of bacterial targets, meaning that a significant number of simultaneous mutations would be needed for the bacteria to protect themselves (50, 52). Silver is particularly attractive for coating medical devices because it adheres well to various materials, is active against a broad spectrum of pathogens, is less prone to resistance, and has shown minimal local or systemic toxicity (51).

Previous *in vitro* studies on bacterial type strains (52–56), have been useful, but resistance levels of persistent clinical isolates may differ from those observed in type strains. Accordingly, we performed an *in vitro* assessment of the effect of silver-coated silicone on bacterial survival and showed that its activity was very high against all tested bacteria. Previous studies on silver-coated endotracheal tubes have demonstrated a 99% reduction in *P. aeruginosa* colonization when compared against untreated tubes, a significant reduction in methicillin resistant *S. aureus* burden (30), and reduced mortality in patients with ventilator-associated pneumonia (57). Silver has also proven effective in managing multidrug-resistant organisms in wound care (58). Our findings support these results, with high susceptibility of even MRSA isolates to silver, although slightly lower than the observed in *P. aeruginosa*. Thus, silver-coated stents may help avoid the problems encountered when treating multidrug-resistant bacteria. Although *S. aureus* isolates were slightly more resistant to silver than *P. aeruginosa*, bacterial death rates of 77–88% represents good activity given the known difficulties in eradicating these microorganisms from colonized patients. Previous studies already suggest lower susceptibility for Gram positive pathogens, possibly due to the difference in thickness of the peptidoglycan layer (30, 59).

Silver is active against a wide range of microorganisms, including bacteria, fungi, and viruses (52) and has a wide range of uses, from slow-release nano-silver linings in cleaning machines (56), to clinical applications in topical creams, wound dressings and coatings for catheters or other medical devices (50). We have shown that silver-coated silicone was active against two of the most problematic pathogens, proving high *in vitro* susceptibility of both Gram-negative and Gram-positive bacteria to silver-coated silicone.

In conclusion, tracheobronchial stent modification with a silver coating is feasible and effective. This could be an important step toward preventing, or at least reducing, stent-related infections. Studies in animal models and larger prospective clinical trials are required in humans to assess the true effect of silver-coated silicone stents on bacterial colonization in the long term.

## Data Availability

The raw data supporting the conclusions of this article will be made available by the authors, without undue reservation.

## Footnotes

1
http://imagej.nih.gov
