# Supplementary material for: Silver-coated silicone stents as an approach to prevent bacterial colonization of central airways after tracheobronchial stenting
Source: Front Microbiol. 2025 Dec 3;16:1713806. doi: 10.3389/fmicb.2025.1713806 (PMC12709627; doi:10.3389/fmicb.2025.1713806)
Supplement: Supplementary file 2 [file Data_Sheet_1.pdf]

Silver-coated silicone stents as an approach to prevent bacterial colonization of central airways after tracheobronchial stenting

Rosa Lopez-Lisbona, Laura Calatayud, Joan Gilabert, Marta Diez-Ferrer, Salud Santos
Salvador Borros, Carmen Ardanuy, Antoni Rosell, Sara Marti.

Supplementary Material

SUPPLEMENTAL METHODS

Procedure for silver-coated silicone

Preparation of PDMS films.

Polydimethylsiloxane (PDMS) films were fabricated following the general protocol previously established (39). Briefly, the two components of the Sylgard 184 silicone elastomer kit (Ellsworth Adhesives, Madrid, Spain) were mixed at a 10:1 base-to-curing-agent ratio. The mixture was cast onto a flat glass substrate using a paint applicator to achieve a uniform layer approximately 500 µm thick. Samples were cured at 60 °C overnight to ensure complete crosslinking and then cut into circular disks (21 mm diameter). Prior to surface treatment, the PDMS coupons were cleaned by immersion in a 5% (w/v) SDS aqueous solution, rinsed twice with Milli-Q water, and stored in 70% ethanol. Before use, samples were air-dried under a clean airflow.

Plasma Reactor.

Surface modifications were carried out in a custom-built, stainless-steel RF plasma reactor developed by the GEMAT group (Barcelona, Spain) as previously described (39-40). The system consists of a cylindrical chamber (25.5 cm diameter, 41.6 cm length) equipped with an aluminum RF-powered electrode serving as the sample holder, while the chamber walls act as the grounded electrode. The RF generator operates at 13.56 MHz through a matching network. Gas and monomer flows are precisely regulated by needle valves connected to a standard manifold, and pressure is monitored via a vacuum gauge controller (MKS PDR900) coupled to a dual cold cathode/micropirani transducer (MKS 972 DualMag). The pumping system includes a nitrogen cold trap and an activated-carbon chemical trap to retain unreacted monomer before it reaches the vacuum pump (Trivac D 16BCS/PFPE, Leybold). The base pressure prior to each experiment was

approximately 6×10^{-4} mbar, and plasma polymerizations were performed at a working pressure of 0.02–0.04 mbar with penta-fluorophenyl methacrylate (PFM) vapor (Apollo Scientific Ltd, Stockport, U.K).

Plasma Polymerization.

Prior to polymerization, the reactor was cleaned by running an O_2/Ar plasma (1:1) (Abelló Linde S.A, Barcelona, Spain) in continuous mode at 150 W for 1 h. PDMS samples were then placed planar to the central aluminum electrode, ensuring that only the upper surface was exposed to the plasma and therefore modified. PFM vapor was introduced into the reactor chamber, and after stabilization of the monomer flow, the plasma polymerized penta-fluorophenyl methacrylate (pp-PFM) process was performed using pulsed plasma at 15 W (duty cycle 10 ms on / 10 ms off) for 5 min. After the plasma was turned off, the monomer flow was maintained for an additional 3 min to complete deposition. The resulting pp-PFM-coated films were stored under an argon atmosphere until further use.

Silver Deposition

Metallic silver was generated in situ on the pp-PFM-modified PDMS surfaces using a Tollens-type reduction reaction (41), adapted from previous work (39-40). The process involves the complexation of silver ions with ammonia, followed by reduction through a mild carbohydrate-based reducing agent.

$$2[Ag(NH_3)_2]^+_{(aq)} + RCHO + 3OH^- \rightarrow 2Ag + RCOO^- + 4NH_3 + 2H_2O$$

Briefly, 1 M glucosamine hydrochloride solution was prepared in Milli-Q water and adjusted to pH 7.4 with concentrated sodium hydroxide. PDMS films were immersed in this solution for 6 h to allow glucosamine attachment to the activated surface. Tollens' reagent was freshly prepared by adding 25 µL of 15% ammonium hydroxide to 1 mL of

0.1 M silver nitrate, forming a transient silver oxide precipitate that redissolves upon addition of another 25 µL of ammonia. The glucosamine-treated samples were washed with Milli-Q water and placed in 4 mL of Milli-Q water, mixed with 1 mL of the Tollens' reagent, and heated at 90 °C for 60 min to promote silver reduction and deposition. The formation of a thin metallic layer was visually confirmed by the characteristic color change of the surface.

Quantification of Surface Silver

To determine the total silver content, the metallic coating was completely dissolved in concentrated nitric acid (Sigma-Aldrich, Burlington, Massachusetts, United States), and the resulting solution was diluted with Milli-Q water prior to analysis. The silver concentration was quantified using inductively coupled plasma–optical emission spectroscopy (ICP–OES, Optima 2100 DV, PerkinElmer) (39-40).

Bacterial viability on silver-coated slides

Confocal microscopy.

After incubation, the slides were removed, washed seven times by immersion in distilled water to eliminate non-adhered bacteria, and dried by capillary action on filter paper. The slides were placed in clean 24-well microtiter plates and stained for 15 minutes in the dark with the fluorescent Live/Dead® BacLight™Bacterial Viability Kit (Life Technologies, Madrid, Spain), following the manufacturer's instructions. Samples were washed three times by immersion in distilled water to remove nonspecific staining and fluorescence was observed by confocal laser microscopy. Images of the double-labeled sections were acquired using a Leica TCS-SL filter-free spectral confocal laser-scanning microscope (Leica Microsystems, Mannheim, Germany) equipped with a 488 nm argon laser, 543 nm and 633 nm He/Ne lasers (*Centres Científics i Tecnològics-Campus de*

Bellvitge, Universitat de Barcelona, Spain) using a 63× magnification oil immersion objective (1.4 numerical aperture), and an image resolution of 1024 × 1024 pixels. The images were acquired randomly from the slide surfaces within 10 min of the staining process to prevent diffusion of the fluorescent dyes and analyzed using the Leica Confocal Software 2.5 (Leica Microsystems). The bacterial adhesion assays were performed in duplicate and on at least two different days.

Images were separated into the two colour channels (green and red) and the percentage of viable and non-viable bacteria calculated with the ImageJ software (U.S. National Institutes of Health, Bethesda, Maryland, USA, http://imagej.nih.gov/ij/, 1997-2014) and the statistical analysis was performed with the GraphPad Prism 5 software (GraphPad Software, Inc., California, USA).
